# The Floating Hospital Association

**Published:** 1884-11

**Authors:** Jos. Stockton, C. L. Rutter

**Affiliations:** President; Chicago, Ill.; Secretary; Chicago, Ill.


CHICAGO, Ill., September 16, 1884.

*To the Editors of the* CHICAGO MEDICAL JOURNAL AND EXAMINER:

The Floating Hospital Association, in making their annual report, desire to thank those who have so generously provided the means for their summer work.

The first boat was sent out to the pier on Monday, July 7th, and the season was continued to and included Friday, September 12th, a period of ten weeks—the longest in our history; 21,489 persons were cared for during this time.

$1,761.71 have been received, $1.428.00 have been expended, $333.71 we have now on hand, which it is proposed to use in the purchase of new hammocks, and other necessaries, made imperative by the very decided increase in the attendance from year to year. It was feared at one time during the summer that we might not be able to go on, but the very liberal support given us by members of the Board of Trade and other constant friends, placed the matter beyond doubt. No accident of any kind occurred, and nothing has marred the success of the work. For ice and milk we are indebted, as we have always been, to the kindness of the respective dealers.

JOS. STOCKTON, President.

C. L. RUTTER, Secretary.

*List of Donations other than Cash, Season of 1884.*—Coal, Robert Law; ice, Washington Ice Co., A. S. Piper & Co., E. A. Shedd & Co., H. P. Smith & Co.; crackers, for the whole season, H. H. Kohlsaat; milk, Kee & Chapell, J. J. Solon, William C. Hill, Duff Brothers, D. McCarthy, —— Fisher, W. Adams; sundries, Edwin Heywood.

